# Farrerol Attenuates Cisplatin-Induced Nephrotoxicity by Inhibiting the Reactive Oxygen Species-Mediated Oxidation, Inflammation, and Apoptotic Signaling Pathways

**DOI:** 10.3389/fphys.2019.01419

**Published:** 2019-11-26

**Authors:** Ning Ma, Wei Wei, Xiaoye Fan, Xinxin Ci

**Affiliations:** ^1^Institute of Translational Medicine, The First Hospital, Jilin University, Changchun, China; ^2^Department of Urology, The First Hospital, Jilin University, Changchun, China

**Keywords:** farrerol, cisplatin, acute kidney injury, reactive oxygen species, Nrf2, oxidative stress, inflammation, apoptosis

## Abstract

Cisplatin is a chemotherapy drug that is often used in clinical practice, but its frequent use often leads to nephrotoxicity. Therefore, we urgently need a drug that reduces the nephrotoxicity induced by cisplatin. Farrerol reportedly has antioxidant potential, but its renal protective effects and potential mechanisms remain unclear. In this study, we used both cell and mouse models to determine the mechanism of farrerol in cisplatin-induced nephrotoxicity. The *in vitro* experiments revealed that farrerol improved cisplatin-induced nephrotoxicity and reactive oxygen species (ROS) production *via* nuclear factor erythrocyte 2-related factor 2 (Nrf2) activation. Moreover, farrerol effectively activated Nrf2 and subsequently increased the expression of Nrf2-targeted antioxidant enzymes, including heme oxygenase-1 (HO-1) and NAD(P)H quinone oxidoreductase-1 (NQO1), but inhibited Kelch-like ECH-associated protein 1 (Keap1) and NADPH oxidase type 4 (NOX4). Furthermore, farrerol attenuated the phosphorylation of C-Jun N-terminal kinase (JNK), extracellular signal-regulated kinase (ERK), and p38 mitogen-activated protein kinase (p38); the activation of phosphorylated nuclear factor-κB (p-NF-κB) and nucleotide-binding domain (NOD)-like receptor protein 3 (NLRP3); and the expression of phosphorylated p53 (p-p53), Bax, and cleaved caspase-3. *In vivo*, farrerol significantly improved cisplatin-induced renal damage, as demonstrated by the recovery of blood urea nitrogen (BUN), serum creatinine (SCr), kidney injury molecule-1 (KIM-1), neutrophil gelatinase-associated lipocalin (NGAL), and pathological damage. Moreover, farrerol inhibited inflammatory and apoptotic protein expression *in vivo*. Notably, farrerol exerted slight protection in Nrf2-knockout mice compared with wild-type mice. These findings indicate that farrerol can effectively activate Nrf2 and can serve as a therapeutic target in the treatment of acute kidney injury (AKI).

## Introduction

Cisplatin is an inorganic platinum chemotherapy drug that is commonly used in clinical chemotherapy treatment (Yang et al., 2018) because it can effectively treat a wide range of solid tumors, including lung, ovarian, and bladder cancers. However, cisplatin has many serious side effects, such as myelosuppression, nephrotoxicity, and ototoxicity (Stojic et al., 2018), and these toxic side effects limit the clinical application of cisplatin as part of standard cancer treatment. The kidney is considered the main pathway for cisplatin excretion and the main site of its accumulation (Arany and Safirstein, 2003). Cisplatin is heavily absorbed by proximal tubular cells, and this absorption leads to renal pathophysiological disorders (Pan et al., 2009). Although the pre-hydration effects of diuretics in patients during chemotherapy reduce the nephrotoxicity of cisplatin, the incidence of cisplatin nephrotoxicity remains high, with an incidence of approximately one-third (Pabla and Dong, 2008). At present, AKI treatment is mainly supportive, and there is no treatment with curative effects (Bagshaw and Wald, 2016). The current diagnostic criteria include the BUN and SCr levels, which primarily reflect the late manifestations of the disease, and thus, early intervention is critical for the effective treatment of AKI (Moledina and Parikh, 2018; Fu et al., 2019).

The generation of ROS is induced after cisplatin enters renal tubular cells, and the levels of generated ROS exceeds the level that can be scavenged by the body, resulting in an imbalance in the redox system (Kim et al., 2019). Excessive ROS can cause oxidative stress, increase the malondialdehyde (MDA) and myeloperoxidase (MPO) levels, and inhibit antioxidant enzymes such as superoxide dismutase (SOD) and glutathione (GSH) (Ma et al., 2015). NADPH oxidase can produce many free radicals, and NOX4 is abundantly produced and leads to excessive ROS production; thus, cisplatin induces significant damage to renal tubular epithelial cells (Ma et al., 2017; Meng et al., 2018b). Nrf2 is a transcription factor that plays a decisive role in the regulation of cellular oxidative defense and redox homeostasis (Li et al., 2018a). In general, Keap1 is an inhibitory molecule that promotes Nrf2 ubiquitination by binding to Nrf2 in the cytoplasm. The activation of Nrf2 enhances the activity of the antioxidant response element (ARE) promoter and thereby simultaneously upregulates the expression of HO-1 and NQO1 and downregulates NOX4 and Keap1 to attenuate cisplatin-induced kidney damage (Babelova et al., 2012; Sedeek et al., 2013). In addition, Nrf2-deficient mice are more vulnerable to oxidative damage than normal mice and also exhibit increased susceptibility to diabetic nephropathy (Jiang et al., 2010), lupus nephritis (Chin et al., 2018), and ischemic AKI (Liu et al., 2009).

Stimulation of the renal tubules by cisplatin significantly increases the production of ROS and thereby the activation of a series of signaling proteins, including JNK (Jaiman et al., 2013), ERK (So et al., 2007), and p38 (Ma et al., 2017). In addition, cisplatin can aggravate AKI by inducing the phosphorylation of ERK and p38 and thereby upregulating the phosphorylation of NF-κB and p53 (Okada et al., 2014; Buendia et al., 2016; Chen et al., 2019). NF-κB, a heterodimer composed of two DNA-binding subunits, namely, p50 and p65, activates the NLRP3 inflammasome and mediates the inflammatory response (Ke et al., 2018). Notably, the mitochondrial pathway underlying the p53-medaited induction of apoptosis can be regulated *via* Bax (a pro-apoptotic protein) and Bcl2 (an anti-apoptotic protein) (Jiang et al., 2009; Okada et al., 2014). Excessive amounts of Bax protein can result in increased binding of Bax to the mitochondrial membrane, and this binding induces the release of cytochrome C from the mitochondria and activates caspase-3, which eventually results in the acceleration of apoptosis (Wei et al., 2007; Ma et al., 2017). Accordingly, Bcl2 can stabilize the mitochondrial membrane potential through a series of inhibitory effects. Consistently, Bcl2 enhances the mitochondrial membrane potential by inhibiting the release of Bax and caspase-3 and thereby blocking the mitochondrial pathway of apoptosis (Arany et al., 2004; Li et al., 2019). Therefore, apoptosis and inflammatory pathways can also be inhibited through the inhibition of ROS production. In addition, Nrf2-targeting molecules provide a new strategy for the treatment of kidney disease.

As major Nrf2 activators, some natural products counteract oxidative stress by modulating the Nrf2/ARE signaling pathway. Farrerol, which was isolated from azaleas, is a novel 2,3-dihydroflavonoid (Wang et al., 2019). We previously showed that farrerol has anti-inflammatory, antibacterial, and antioxidant properties (Ci et al., 2012; Liu et al., 2015; Wang et al., 2019) and that farrerol can activate Nrf2 in RAW 264.7 cells to resist oxidative stress (Ci et al., 2015). Thus far, the protective effect of farrerol on cisplatin-induced AKI has not been reported. Here, we used both cellular and mice models to study the effects and underlying mechanisms of farrerol on cisplatin-induced nephrotoxicity.

## Materials and Methods

### Reagents and Chemicals

Farrerol was purchased from Chengdu Pufei De Biotech Co., Ltd. (Chengdu, China). Anti-phosphorylated c-Jun NH2-terminal kinase (JNK), β-actin, and NOX4 antibodies were obtained from Sungene Biotech Co., Ltd. (Tianjin, China) and Abcam (Cambridge, MA, USA). Primary antibodies against Nrf2, Keap1, HO-1, NQO1, P53, caspase-3, Bax, Bcl2, phospho-JNK, phospho-ERK, phospho-p38, and NF-κB were purchased from Abcam (Cambridge, MA, USA) and Cell Signaling (Boston, MA, USA). Phosphatase p53 was purchased from ImmunoWay. KIM-1- and NGAL-specific antibodies were purchased from R&D Systems, and the BCA protein assay kit (Beyotime, China) was used to evaluate the protein concentrations. The cell culture medium DMEM, antibiotic-antimycotic, and trypsin-EDTA were purchased from Corning, MBI, and Biofil, respectively. Dimethyl sulfoxide (DMSO) and DCFH-DA were purchased from Sigma Chemical Co. (St. Louis, MO, USA). In addition, BUN, SCr, MDA, MPO, GSH, and SOD detection kits were obtained from Nanjing Jiancheng Bioengineering Institute (Nanjing, China).

### Cell Culture and CCK-8 Analysis

Mouse tubular epithelial cells (MTECs) and human proximal tubule cells (HK-2) were purchased from the Chinese Cell Bank (Beijing, China). MTECs were cultured in DMEM containing 10% fetal bovine serum (FBS), 100 U/ml penicillin, 100 U/ml streptomycin, and 3 mM glutamine at 37°C in an environment with 95% air and 5% carbon dioxide. We used the CCK-8 assay kit to detect viability of the cells. MTECs and HK2 were seeded in 96-well plates (1.5 × 10^4^ cells/well), and after 24 h of culture, the cells were treated with farrerol and cisplatin for 18 h and incubated with 10 μl of CCK-8 reagent for 2 h in an incubator at 37°C. The absorbance at 450 nm was then measured to determine the cell viability.

### Intracellular Reactive Oxygen Species Measurement

The active oxygen scavenging activity of farrerol was determined using the oxidant-sensitive fluorescent probe DCFH-DA. MTECs were plated in 96-well plates (1.5 × 10^4^ cells/well), pretreated with or without farrerol (5, 10, and 20 μM) for 18 h, and then stimulated with cisplatin (20 μM) for 30 min. The cells were then incubated with 50 mM DCFH-DA for 20 min. The fluorescence intensity was measured with an excitation wavelength of 485 nm and an emission wavelength of 535 nm.

### Quantitative Real-Time Polymerase Chain Reaction

The mRNA expression levels of Nrf2 in MTECs and HK2 were detected by qPCR. Total RNA was extracted from the cells using the TransGen Biotech (Beijing, China) reagent according to the manufacturer’s instructions. EasyScript First-Strand cDNA Synthesis SuperMix was used to obtain the cDNA templates (TransGen Biotech, Beijing, China), and mRNA transcription was determined using SYBR Green qPCR kits and analyzed using real-time PCR systems. Each analysis was repeated three times.

### Hoechst 33342/PI Double Staining

MTECs were seeded in 12-well plates (3 × 10^5^ cells/well) and cultured for 24 h, and the medium was then aspirated and replaced with low-sugar serum-free DMEM. The cells were pretreated with farrerol for 1 h prior to stimulation with cisplatin for 18 h. We subsequently added 2 μl of Hoechst 33342 solution and 6 μl of PI staining solution, heated the cells in a warm water bath at 37°C for 15 min, and examined the cells by fluorescence microscopy.

## Experimental Design and Animal Procedures of Acute Kidney Injury

An animal model of cisplatin-induced AKI was established in male C57BL/6 mice. The mice were randomized into the following four groups (*n* = 10/group): control (saline), farrerol (20 mg/kg), cisplatin only (20 mg/kg), and cisplatin (20 mg/kg) + farrerol (20 mg/kg). Prior to the *in vivo* experiments, all the mice were starved for 12 h. After fasting, the mice belonging to the cisplatin (20 mg/kg) + farrerol (20 mg/kg) group were treated with 20 mg/kg farrerol and administered 20 mg/kg cisplatin *via* intraperitoneal injection1 hour later. Farrerol was re-administered 24 and 48 h after the cisplatin injection, and the mice were sacrificed 24 h after the last dose of farrerol. C57BL/6 wild-type mice were purchased from Liaoning Changsheng Technology Industrial Co., Ltd. (Certificate SCXK2010-0001; Liaoning, China), and C57BL/6 Nrf2-knockout mice were purchased from The Jackson Laboratory (Bar Harbor, ME, United States). All the animals were housed in a specific pathogen-free facility, and this experiment was approved by the Animal Health and Research Ethics Committee of Jilin University.

### Histopathology Assessment

Paraffin-embedded mouse kidneys were prepared and cut into 3-μm-thick sections. The degree of representativeness of each histological sections of kidney tissue was determined by H&E staining (Huang et al., 2017). Histological damage was determined according to necrosis, cast formation, brush border loss, tubular degeneration, and vacuolization. Tissue scores are indicated by damaged tubules. Scored as a percentage of 0, 1, 2, 3, and 4, indicating damage to tissues of normal, 1–25%, 26–50, 51–75, and ≥ 75%, respectively (Wang et al., 2018).

### Blood Measurements

The renal function was measured based on the BUN and SCr levels. Blood samples were collected from the mice immediately after sacrifice. The levels of BUN and SCr in the blood samples were measured using kits purchased from Nanjing Jiancheng Bioengineering Institute (Nanjing, China) according to the instructions provided with the kits.

### Measurement of the Myeloperoxidase, Malondialdehyde, Glutathione, and Superoxide Dismutase Levels in Kidney Tissues

All the mice were sacrificed by anesthesia with ether, and the bilateral kidneys were excised 3 days after stimulation with cisplatin. The kidney tissue was homogenized and dissolved in extraction buffer for analysis of the MPO, MDA, SOD, and GSH levels. To examine the neutrophil accumulation and the lipid peroxidation levels in kidney tissue, the MPO and MDA levels were assessed using commercially available assay kits (Nanjing Jiancheng Bioengineering Institute, China) according to the manufacturer’s instructions. In addition, to further measure the antioxidant enzyme activity in lung tissue, the SOD and GSH levels were measured using kits (Keygen Biotech. Co., Ltd., Nanjing, China) according to the manufacturer’s instructions.

### Western Blot Analysis

Renal tissue and MTECs were collected and lysed on ice for 30 min, and the protein concentrations were analyzed using a BCA Protein Assay Kit. Equal amounts of protein were separated on 10 or 12.5% SDS-polyacrylamide gels and transferred to PVDF membranes. The membranes were blocked in 5% nonfat milk on a shaker for 1 h at room temperature, incubated with the corresponding primary antibodies overnight at 4°C, and probed with the corresponding secondary antibody at room temperature. The bands were then observed using ECL, and the band intensities were quantified using ImageJ gel analysis software.

### Statistical Analysis

All the above-mentioned data are expressed as the means ± SEMs and were analyzed using SPSS 19.0 (IBM). The experimental data were compared by one-way analysis of variance (ANOVA). Statistical significance was defined as *p* < 0.05.

## Results

### The Effects of Farrerol on Cisplatin-Induced Reactive Oxygen Species Depend on Nuclear Factor Erythrocyte 2-Related Factor 2

**Figure 1:**
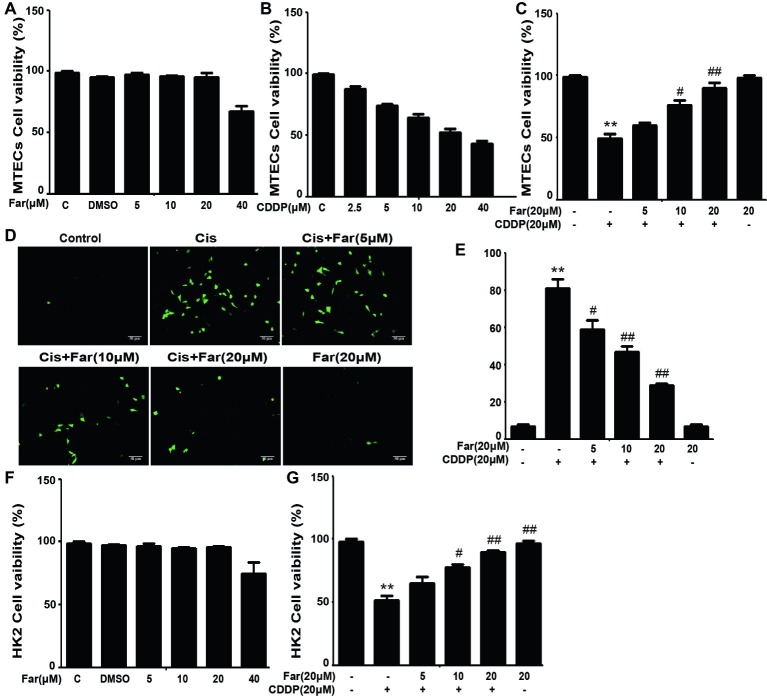
Farrerol affects cisplatin-induced oxidative stress by inhibiting ROS production *in vitro*. MTECs were treated with the specified concentration of farrerol **(A)** or cisplatin **(B)** for 18 h, and the cell viability was then determined through the CCK-8 assay. **(C)** MTECs were treated with various concentrations of farrerol (5, 10 and 20 μM) for 1 h and then with cisplatin (20 μM) for 18 h, and the CCK-8 assay was then used for the assessment of cell viability. MTECs were stained with 50 mM ROS fluorescent probe for 30 min, and the resulting fluorescence was detected using a fluorescence microscope **(D)** and multiple detection readers **(E)**. **(F,G)** HK2 cells were treated as in **(A)** and **(C)**, and the cell viability was measured with the CCK-8 assay. All of the data displayed represent the mean of three independent experiments. *p < 0.05 and **p < 0.01 vs. the control group; ^#^*p* < 0.05 and ^##^*p* < 0.01 vs. the CDDP group.

**Figure 2:**
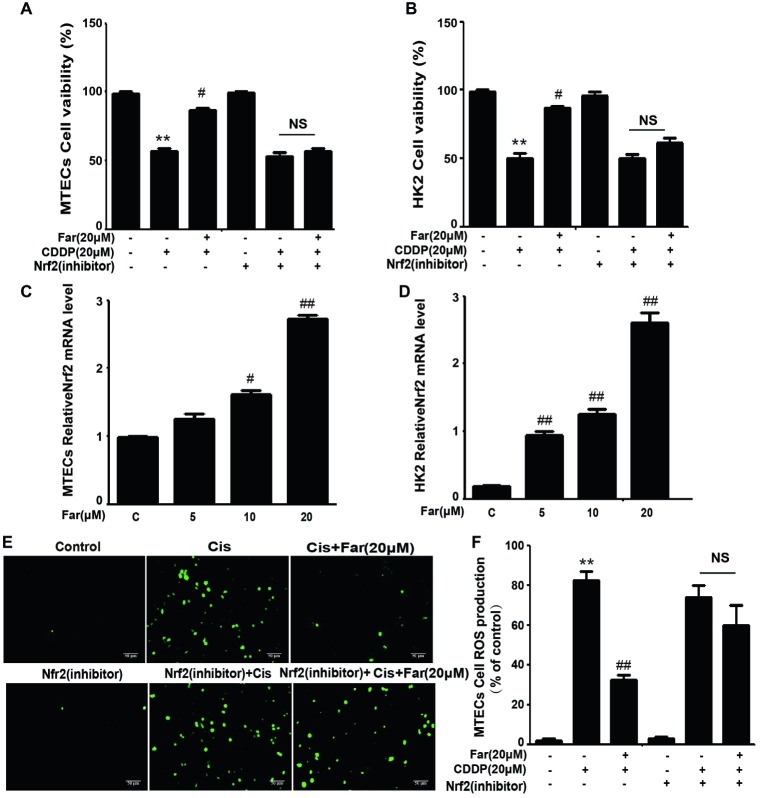
The effects of farrerol on ROS production depend on Nrf2 *in vitro*. Effect of farrerol on MTECs **(A)** and HK2 cell **(B)** viability in the presence or absence of a Nrf2 inhibitor. Nrf2 mRNA expression in MTECs **(C)** and HK2 **(D)** treated with different concentrations of farrerol (5, 10, and 20 μM). **(E)** The production of ROS by MTECs was examined using the same protocol shown in Figure 1D, with or without pretreatment with a Nrf2 inhibitor. **(F)** Quantitative and statistical analysis of ROS-positive cells. All experiments were repeated three times. *p < 0.05 and **p < 0.01 vs. the control group; ^#^*p* < 0.05 and ^##^*p* < 0.01 vs. the CDDP group. NS, no specificity.

To identify the signals involved in the effects, we initially focused on ROS, which are associated with cisplatin nephrotoxicity. We first observed the cytotoxic effects induced by cisplatin and the protective effects of farrerol using the CCK8 method. As shown in Figure 1A, farrerol (0–20 μM) did not exert cytotoxicity in MTECs. Furthermore, cisplatin reduced the viability of the cells after culture for 18 h with an IC_50_ of 20 μM (Figure 1B). Based on these data, MTECs were pretreated with farrerol (0–20 μM) for 1 h and then incubated with 20 μM cisplatin for 18 h. The farrerol pretreatment, particularly at a concentration of 20 μM, attenuated the cytotoxicity of cisplatin (Figure 1C). In addition, a fluorescence microscopy analysis showed that high levels of ROS were produced after cisplatin treatment, but farrerol (20 μM) pretreatment significantly reduced the production of ROS (Figures 1D,E). In addition, the results obtained from the treatment of HK2 cells with farrerol and cisplatin were consistent with the previous results (Figures 1F,G). Nrf2 is an important regulator of cell resistance to oxidants. Specifically, Nrf2 controls the baseline and induced expression of a range of antioxidant response element-dependent genes to regulate the damage caused by oxidant exposure (Ma, 2013). To investigate whether the protective effect of farrerol on cisplatin-induced cytotoxicity is related to Nrf2, we investigated the influence of Nrf2 inhibitors on this process through a CCK-8 assay. The experimental results suggested that the mechanism through which farrerol protects against CDDP cytotoxicity might be related to the activation of Nrf2 expression (Figures 2A,B). As shown in Figures 2C,D, a qPCR analysis showed that Nrf2 mRNA expression was markedly induced *in vitro* by treatment with different concentrations of farrerol. To further confirm that farrerol inhibited ROS production by activating Nrf2, we subsequently inhibited cisplatin nephrotoxicity and found that inhibitors of Nrf2 effectively inhibited the protective effect of farrerol against cisplatin and eliminated the farrerol-induced clearance of ROS (Figures 2E,F).

### Farrerol Attenuates Cisplatin-Induced Oxidation and Inflammation *in vitro*

**Figure 3:**
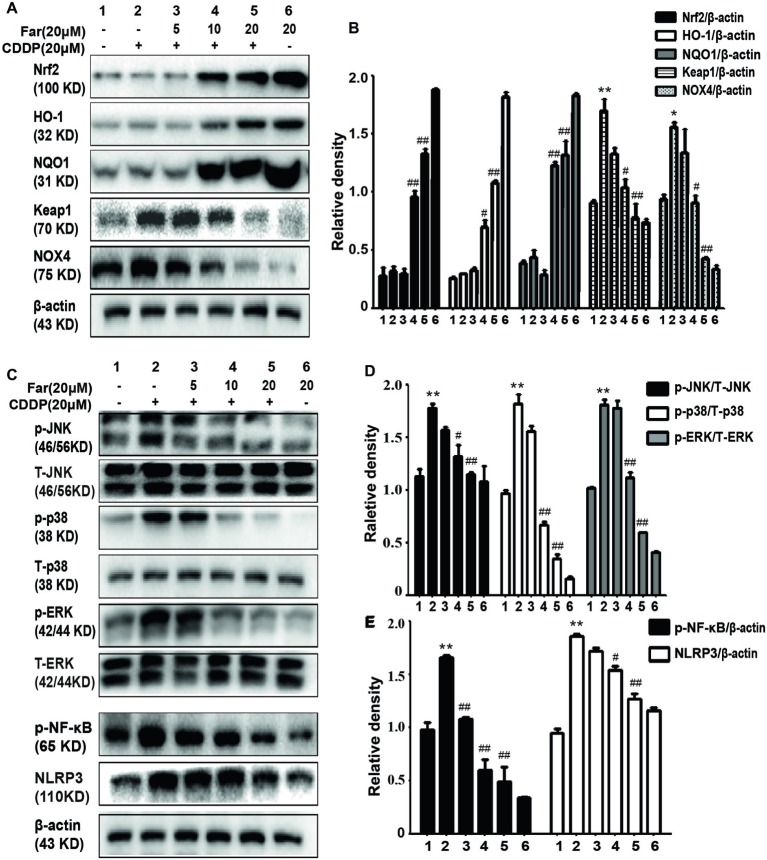
Effects of farrerol on cisplatin-induced oxidation and inflammation pathway *in vitro*. MTECs were treated with different concentrations of farrerol (5, 10, or 20 μM) for 1 h and then with cisplatin (20 μM) for 18 h, and whole cell lysates were collected for Western blotting. **(A,B)** Quantification of the relative Nrf2, HO-1, NQO1, Keap1, and NOX4 protein levels obtained by Western blotting. **(C–E)** Representative Western blots showing the p- JNK/T-JNK, p-ERK/T-ERK, p-p38/T-p38, p- NF-κB (p-p65), and NLRP3 protein levels. All experiments were repeated three times. *p < 0.05 and **p < 0.01 compared with the control group; ^#^*p* < 0.05 and ^##^*p* < 0.01 compared with the CDDP group. β-actin was used as an internal control.

We performed *in vitro* Western blotting analyses to further investigate the potential mechanism of farrerol, and the results indicated that farrerol activated Nrf2 in a dose-dependent manner and enhanced the levels of the downstream proteins HO-1 and NQO1. In contrast, farrerol alleviated the levels of Keap1 and Nox4 in a concentration-dependent manner (Figures 3A,B). These results also confirmed that the protective effects of farrerol on AKI might be related to Nrf2 activation. The mitogen-activated protein kinase (MAPK) pathway plays a crucial role in both inflammation and apoptosis (Okada et al., 2014). Farrerol reduced the cisplatin-induced phosphorylation of JNK, p38, and ERK to inhibit the MAPK pathway (Figures 3C,D) and downregulated the protein expression of p-NF-κB and NLRP3 to inhibit inflammation (Figures 3C,E).

### Farrerol Regulates Cisplatin-Induced Mouse Tubular Epithelial Cells Apoptosis

**Figure 4:**
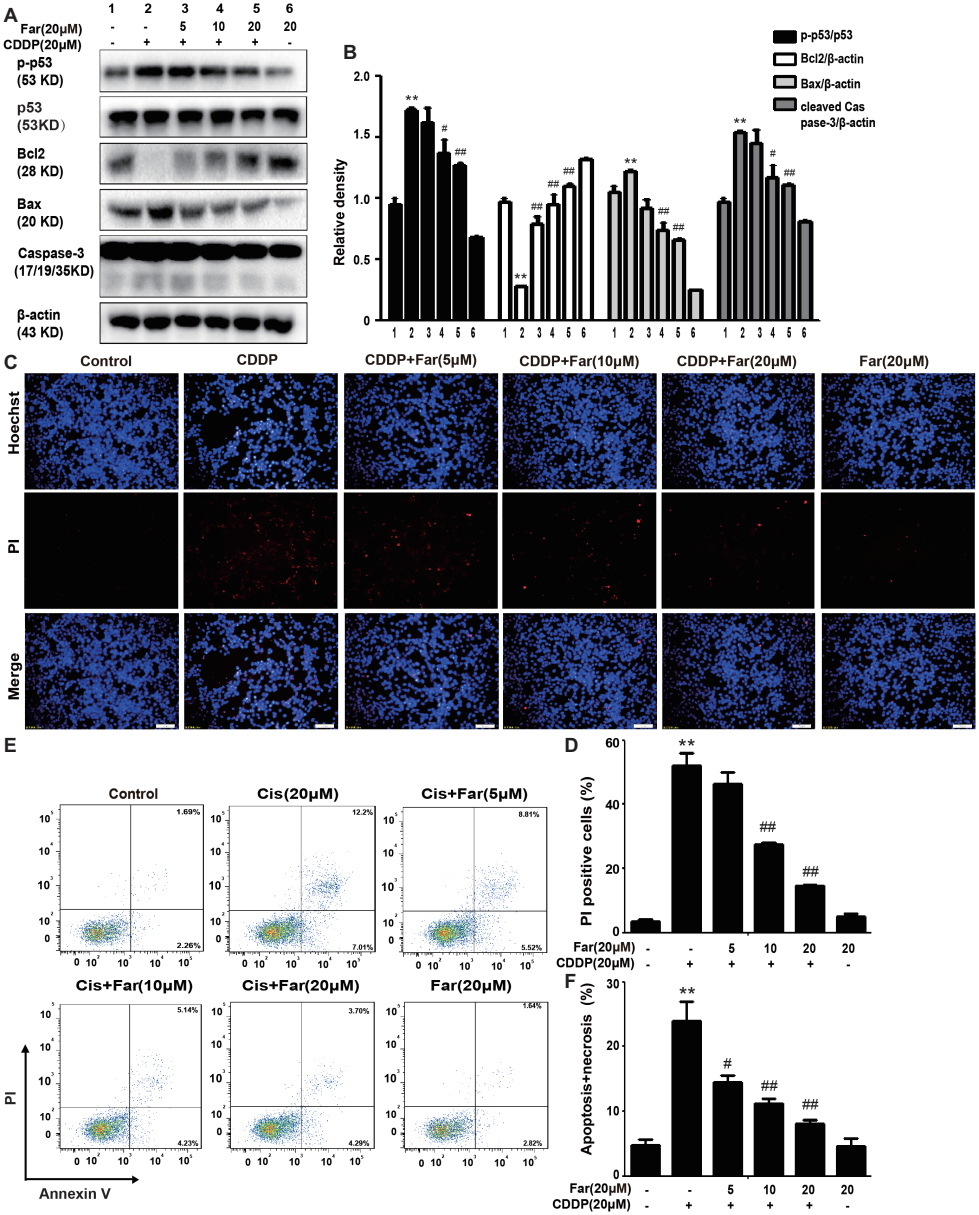
Effects of farrerol on inhibition of the apoptosis of cisplatin-treated MTECs. MTECs were plated in six-well plates, preincubated with farrerol (5, 10, or 20 μM) for 1 h and then stimulated with cisplatin (20 μM) for 18 h. **(A,B)** The expression of the representative proteins p-p53, p53, Bcl2, Bax, and caspase-3 was assessed by Western blotting. **(C,D)** MTECs were stained with Hoechst/PI for 15 min, and the fluorescence was then rapidly detected with a fluorescence microscope. **(E,F)** The percentages of apoptotic and necrotic cells were determined by flow cytometry. All experiments were duplicated three times. *p < 0.05 and **p < 0.01 vs. the control group; ^#^*p* < 0.05 and ^##^*p* < 0.01 vs. the CDDP group. β-actin was used as an internal control.

During activation of the MAPK pathway, ERK and P38 phosphorylation can effectively stimulate p-p53 production to induce apoptosis (Li et al., 2018b), which is consistent with the Western blot results obtained in our study. Pretreatment with farrerol inhibited the expression of p-p53, Bax, and cleaved caspase-3 and activated the expression of Bcl2 in a concentration-dependent manner *in vitro* (Figures 4A,B). In addition, the cells were stained with PI and Hoechst dyes, and their apoptosis was observed using a fluorescence microscope. The results showed that farrerol significantly reduced the number of PI-positive cells and effectively inhibited apoptosis in a dose-dependent manner (Figures 4C,D). Similarly, the same experimental results were obtained by flow cytometric analysis (Figures 4E,F).

### Effects of Farrerol on Cisplatin-Induced Kidney Dysfunction

**Figure 5:**
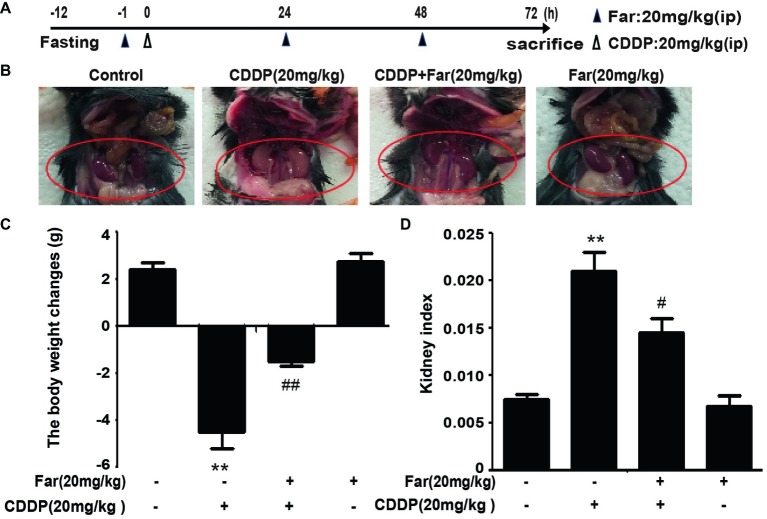
Effects of farrerol on cisplatin-induced AKI. **(A)** Experimental design and schedule for treatment with cisplatin alone or in combination with farrerol. After 12 h of fasting, C57BL/6 mice were treated with farrerol (20 mg/kg) and administered 20 mg/kg cisplatin or vehicle 1 h later *via* intraperitoneal injection. Farrerol was administered 24 and 48 h after injection, and the mice were sacrificed 24 h after the last dose of farrerol. **(B)** Images of kidneys without magnification. **(C)** The changes in body weight were calculated by subtracting the body weight at 72 h from and the body weight 1 h prior to treatment. **(D)** The kidney index is expressed as the weight of the kidney divided by the body weight at 72 h. All the data are presented as the means ± SEM (*n* = 10 per group). All experiments were duplicated three times. *p < 0.05 and **p < 0.01 vs. the control group; ^#^*p* < 0.05 and ^##^*p* < 0.01 vs. the CDDP group.

**Figure 6:**
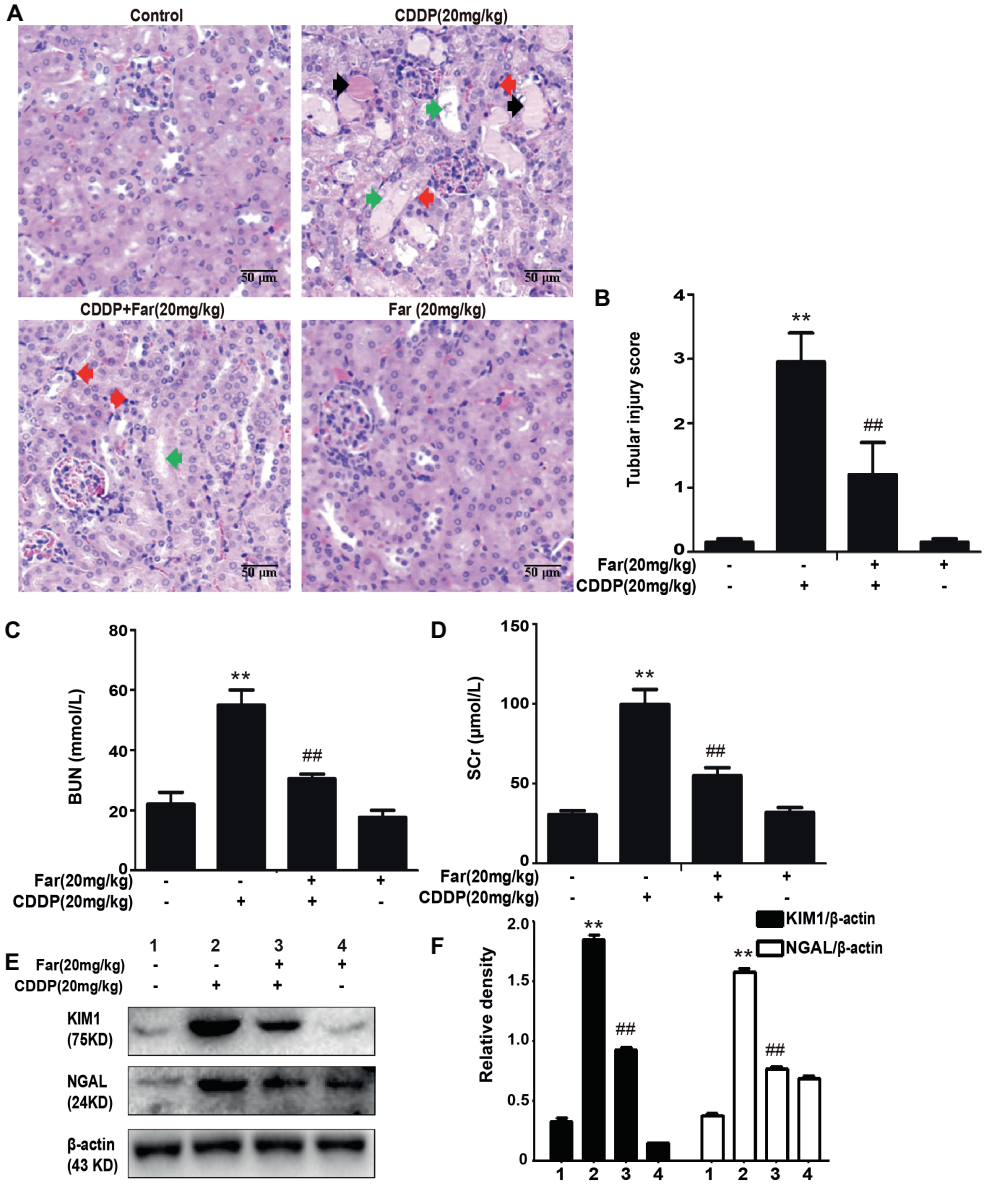
Effects of farrerol on cisplatin-induced kidney dysfunction. **(A)** Representative tissue sections of kidneys stained with H&E. Necrosis (red arrow); Cast formation (black arrow); brush border loss, tubular degeneration, and vacuolization (green arrow). **(B)** Tissue damage score by percentage of damaged tubules (0, no damage; 1, <25% damage; 2, 25–50% damage; 3, 50–75% damage; and 4, >75% damage). The collected blood was used for calculation of the BUN **(C)** and SCr **(D)** levels. Data are presented as means ± SEM (*n* = 10 in each group). **(E,F)** The kidney tissue was homogenized and dissolved in extraction buffer, and Western blotting was performed to evaluate the protein expression of the proximal tubular injury markers, including KIM1 and NGAL. All experiments were repeated three times. *p < 0.05 and **p < 0.01 compared with the control group; ^#^*p* < 0.05 and ^##^*p* < 0.01 compared with the CDDP group.

To determine the effect of farrerol on AKI, we established an experimental model of cisplatin-treated mice (Figure 5A). After 12 h of fasting, the mice received an individual intraperitoneal injection of cisplatin (20 mg/kg) to induce AKI. Morphological observations revealed that the kidneys of the cisplatin group showed significant edema, mainly characterized by an increased kidney volume and a pale kidney color. However, the morphology of the kidneys of the mice belonging to the treatment group was significantly improved (Figure 5B). Furthermore, we measured and analyzed changes in the body weights of the mice, and the results showed that the weights of the stimulated group were significantly decreased. In addition, no evident difference in body weight was found between the farrerol and control groups (Figure 5C). Our analysis of the kidney index also revealed that farrerol significantly improved the cisplatin-induced kidney damage (Figure 5D). To understand the mechanism of action of farrerol, we performed H&E staining and scored the stained sections. Compared with the control group, the cisplatin group showed more morphological damage, including necrosis, cast formation, brush border loss, tubular degeneration, and vacuolization. Notably, these histological lesions were significantly attenuated in the farrerol-treated mice (Figures 6A,B). Consistent with the improvements in the kidney morphology, farrerol significantly reversed the increase in the BUN and SCr levels observed after 72 h of cisplatin treatment (Figures 6C,D). Moreover, farrerol improved the significant upregulation of renal KIM1 and NGAL (proximal tubular injury markers) caused by cisplatin, as shown in Figures 6E,F.

### Farrerol Improves Cisplatin-Induced Oxidative Stress and Inflammation *in vivo*

**Figure 7:**
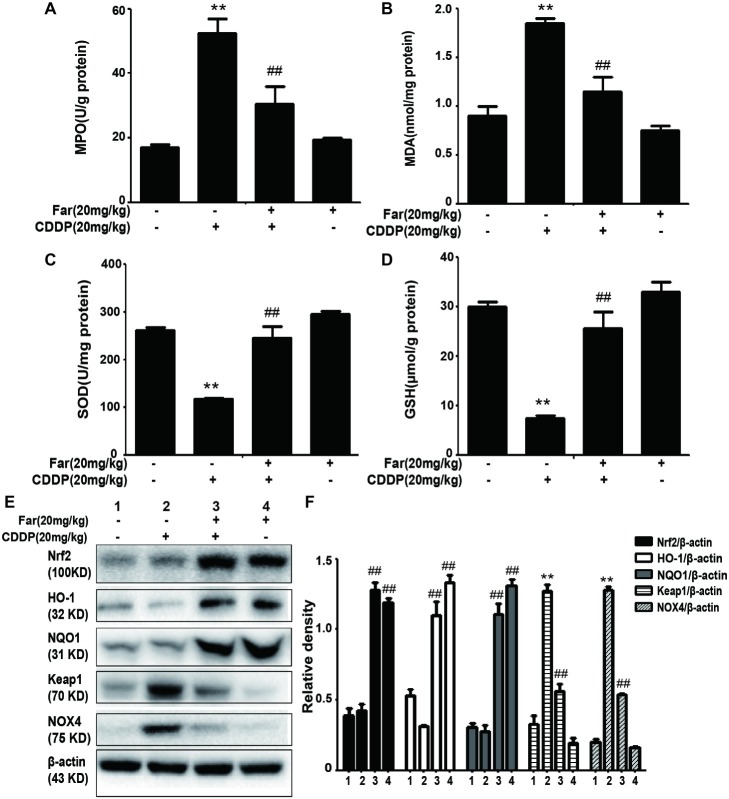
Effects of farrerol on the levels of oxidative pathway-related proteins induced by cisplatin. **(A–D)** Renal tissues were collected from the mice to measure the production of MPO, MDA, SOD, and GSH. Data are expressed as mean ± SEM (*n* = 10 per group). **(E,F)** Representative Western blots showing the effect of farrerol on the levels of oxidative pathway-related proteins (including Nrf2, HO-1, NQO1, Keap1, and NOX4) in mouse kidney tissues. All experiments were duplicated three times. *p < 0.05 and **p < 0.01 vs. the control group; ^#^*p* < 0.05 and ^##^*p* < 0.01 vs. the CDDP group.

Because oxidative stress plays a key role in cisplatin-induced mice, we evaluated the extent of cisplatin-induced oxidative damage by measuring various oxidation markers. The data demonstrate that cisplatin can increase the MPO and MDA concentrations while reducing the SOD and GSH levels. Consistently, we observed that farrerol attenuated cisplatin-induced AKI in mice by reducing the MPO and MDA levels and increasing the SOD and GSH levels compared with the levels obtained with cisplatin treatment only (Figures 7A–D). To assess the antioxidant mechanism of farrerol in a cisplatin-induced AKI model, we extracted protein from kidney tissue and performed Western blotting. The results showed that farrerol efficiently activated Nrf2 and increased the levels of the downstream proteins HO-1 and NQO1. In contrast, farrerol attenuated the expression of Keap1 and NOX4 (Figures 7E,F). These data indicated that the antioxidant ability of farrerol involves activation of the Nrf2/ARE pathway, and this finding was examined further in the subsequent experiments.

### Farrerol Ameliorates Cisplatin-Induced Inflammation and Apoptosis in Mice

**Figure 8:**
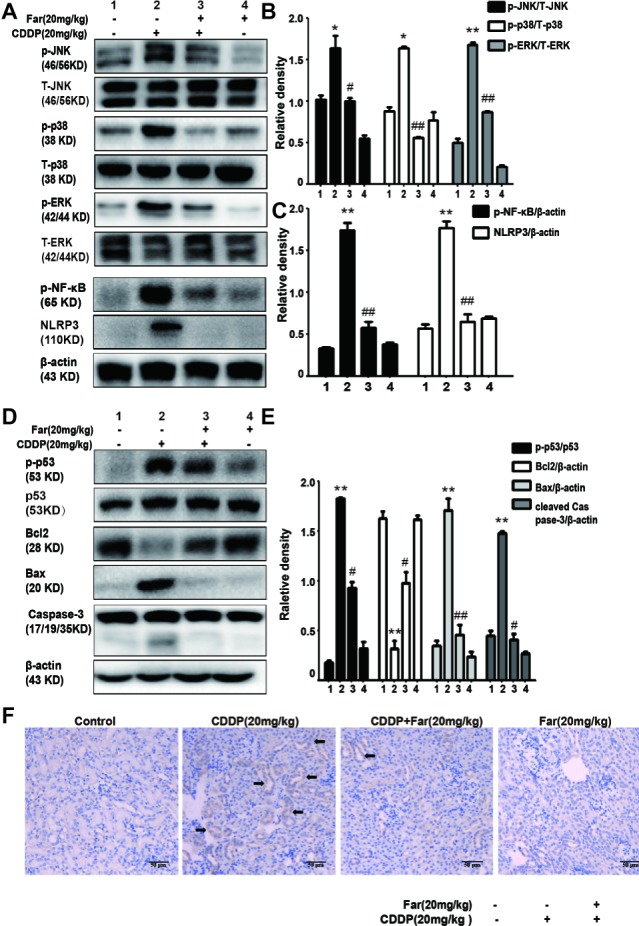
Effects of farrerol on cisplatin-induced inflammation and apoptosis in AKI. **(A–C)** p-JNK / T-JNK, p-ERK / T-ERK and p-p38 / T-p38, p-NF-κB (p-p65) and NLRP3 protein levels were analyzed by Western blot. **(D,E)** Representative Western blot showing the expression of p-p53, p53, Bax, caspase-3 and Bcl2 in the kidney. **(F)** Representative photographs of immunohistochemical analysis of Caspase3 (black arrow) for kidney sections of the control, CDDP, CDDP + Far, and Far groups. Data are presented as the means ± SEM (*n* = 10 per group). All of the data displayed represent the mean of three independent experiments. *p < 0.05 and **p < 0.01 vs. the control group; ^#^*p* < 0.05 and ^##^*p* < 0.01 vs. the CDDP group. NS, no specificity. β-actin was used as an internal control.

We then investigated whether farrerol regulates the MAPK pathway in the AKI model. As shown in Figures 8A,B, the protein expression of p-JNK, p-ERK, and p-p38 was evidently upregulated in the cisplatin group compared with the control group, and farrerol pretreatment effectively reduced the expression levels of these proteins. The MAPK pathway plays a crucial role in both inflammation and apoptosis (Okada et al., 2014). We subsequently investigated the role of farrerol in the inflammatory and apoptotic pathways *in vivo*, and the results indicated that farrerol can inhibit inflammation by inhibiting the expression of p-NF-κB and NLRP3 (Figures 8A,C). In addition, farrerol increased the Bcl2 protein levels compared with the levels obtained with cisplatin treatment alone and reduced the levels of p-p53, Bax, and cleaved caspase-3 (Figures 8D,E). To further confirm this hypothesis, the kidney specimens were subjected to immunohistochemical staining of caspase-3. As shown in Figure 8F, the expression of caspase-3 in the kidney tissues of the mice exposed to CDDP was elevated compared with that found in the control group, and farrerol pretreatment significantly reduced these levels. All the results confirm that treatment with farrerol can improve CDDP-induced inflammation and the apoptotic pathway.

### Nuclear Factor Erythrocyte 2-Related Factor 2 Deficiency Aggravates Cisplatin-Induced Acute Kidney Injury *in vivo*

**Figure 9:**
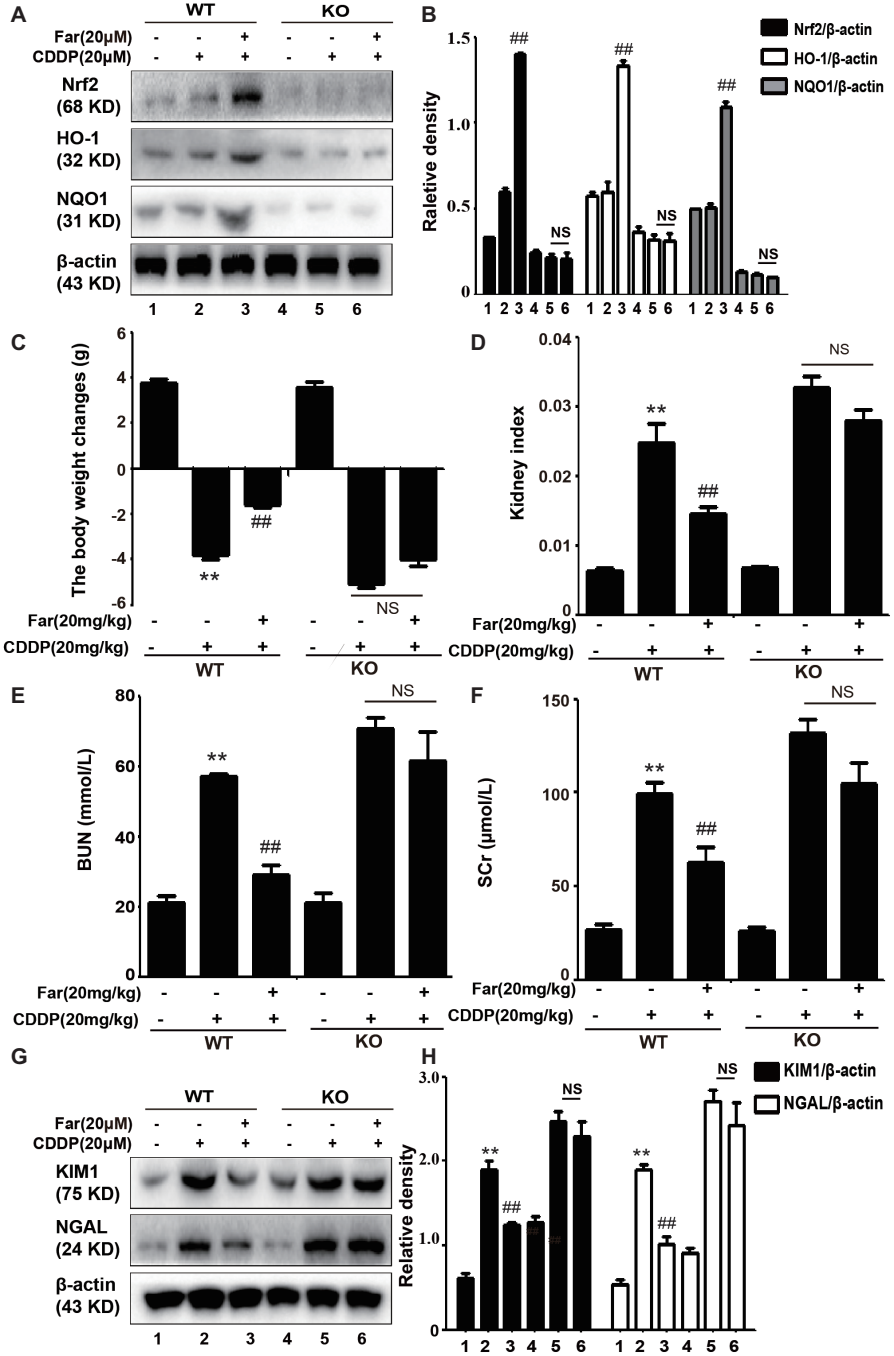
The effects of farrerol on cisplatin-induced AKI depend on Nrf2 *in vivo*. After 12 h of fasting, wild-type and Nrf2-knockout C57BL/6 mice were injected intraperitoneally with farrerol (20 mg/kg), and cisplatin (20 mg/kg) was administered by intraperitoneal injection 1 h later. Farrerol was then administered at 24 and 48 h after the injection, and the mice were sacrificed 24 h after the last farrerol administration. After recording the body and kidney weights, the kidneys and blood from all the tested mice were collected. **(A,B)** Protein expression of Nrf2, HO-1, and NQO1 in the wild-type and Nrf2-knockout mice. The body weight changes **(C)**, kidney injury index **(D)**, BUN **(E)**. and SCr levels **(F)** in the wild-type and Nrf2-knockout mice were measured. All the data are presented as the means ± SEM (*n* = 10 per group). **(G,H)** Western blot analysis of KIM1 and NGAL protein expression in the kidneys. All experiments were duplicated three times. *p < 0.05 and **p < 0.01 compared with the control group; ^#^*p* < 0.05 and ^##^*p* < 0.01 compared with the CDDP group. β-actin was used as an internal control, NS, no specificity; WT, wild-type C57BL/6 mice; KO, Nrf2-knockout C57BL/6 mice.

To test whether the improvements mediated by Nrf2 in CDDP-induced AKI contribute to the protective effects of farrerol, we also induced AKI in wild-type and Nrf2-knockout C57BL/6 mice using a similar approach to that used to obtain the data shown in Figure 4A. The expression of the antioxidant response gene Nrf2 and that of its downstream related genes HO-1 and NQO1 were significantly increased in the wild-type mice, but no significant expression was observed in the Nrf2-knockout mice (Figures 9A,B). We then assessed renal function by measuring the body weight changes, kidney index, and levels of BUN, SCr, KIM1 and NGAL. Compared with the control group, kidney function was significantly reduced in the cisplatin group, and Nrf2 knockout mice have worsened symptoms than the wild-type mice (Figures 9C–H). Notably, the results presented in Figures 9A–H showed that farrerol exerted no obvious protective effect on Nrf2-deficient mice.

## Discussion

Acute kidney injury (AKI) caused by sepsis, ischemia, or nephrotoxic agents is characterized by abrupt and reversible kidney dysfunction (Doi and Rabb, 2016). The pathogenesis of AKI is complex and diverse and is mainly related to oxidative stress, inflammation, and apoptosis. Among these symptoms, nephrotoxicity is a main side effect of cisplatin chemotherapy, and this side effect limits the usage of cisplatin for the treatment of cancer patients (Ozkok and Edelstein, 2014). It has been reported that the entry of cisplatin into the proximal tubules of the kidney induces the production of a large amount of ROS, which might destroy the balance of the body’s redox system (Yen et al., 2019). Some studies have shown that antioxidants can prevent cisplatin-induced nephrotoxicity through their effects on inflammation and apoptosis (Shen et al., 2019), and other studies have shown that antioxidants can improve cisplatin-induced cytotoxicity by inhibiting inflammatory responses and reducing apoptosis (Lin et al., 2019).

Under various pathological conditions, ROS produced by oxidative stress are inseparable from renal damage (Nath and Norby, 2000). The treatment of mice with cisplatin significantly increases the BUN, SCr, KIM1, and NGAL levels (Figures 6C–F) and significantly worsens the histopathology results mainly including necrosis, cast formation, and brush border loss (Figures 6A,B). Therefore, we concluded that ROS-mediated oxidative stress is associated with cisplatin-induced AKI. Farrerol is a novel 2,3-dihydroflavonoid isolated from rhododendron (Wang et al., 2019) that reportedly exerts antioxidant effects (Liu et al., 2015; Wang et al., 2019). The administration of 20 mg/kg cisplatin was adjusted based on the protocol used for farrerol administration (Figure 4A). CDDP-induced oxidative stress is characterized by elevated MDA and MPO levels and decreased SOD and GSH levels (Ma et al., 2015), and these effects were reversed by farrerol in our experiments (Figures 7A–D). Based on these data, farrerol can effectively inhibit oxidative stress, but the specific mechanism remains unclear. Farrerol also reportedly activates Nrf2 to alle*via*te oxidative lesions in RAW 264.7 cells (Liu et al., 2015). Inspired by these observations, we utilized antioxidant molecules to investigate the possible relationship between Nrf2 and antioxidant activity to further explore the pharmacological effects of farrerol. The activation of the Nrf2 pathway plays an important role in the cellular redox status and oxidative stress (Dodson et al., 2015). Notably, a Nrf2 inhibitor abolished farrerol-induced Nrf2 activation and increased cisplatin-induced cytotoxicity, which indicated that farrerol might protect against cisplatin-induced toxicity through Nrf2 activation (Figures 2A,B). In addition, we observed that farrerol failed to attenuate the production of ROS in cisplatin-treated tubular cells in which Nrf2 was inhibited (Figures 2E,F), and these results suggested that farrerol inhibited ROS production by activating Nrf2 and subsequently inhibited cisplatin nephrotoxicity *in vitro*. In addition, qPCR and Western blotting assays were performed to detect the expression of Nrf2, and the results demonstrated that farrerol upregulated the expression of Nrf2 in a dose-dependent manner *in vitro* (Figures 2C,D). Our results also showed that farrerol dose-dependently upregulates the levels of HO-1 and NQO1 proteins and downregulates the expression of Keap1 and NOX4 *in vitro* (Figures 3A,B) and *in vivo* (Figures 7E,F). To support these findings, we used Nrf2-knockout and wild-type mice models to further explore the mechanism through which Nrf2 protects against CDDP-induced AKI. Our findings revealed that the cisplatin group exhibited significantly reduced kidney function (including body weight changes; kidney index; BUN, SCr, KIM1, and NGAL levels) and that Nrf2-knockout mice exhibited lower kidney function compared with the wild-type mice (Figures 9C–H). We also found that farrerol exerted only a slight protective effect on Nrf2-deficient mice, and no significant expression of Nrf2, HO-1, and NQO1 was detected in the Nrf2-knockout mice (Figures 9A,B).

Furthermore, increasing lines of evidence show that ROS produced by cisplatin mediate inflammatory pathways by activating the MAPK and NF-κB pathways (Baker et al., 2011; Li et al., 2014). NF-κB, which is a central regulator of the kidney inflammatory response mechanism (Meng et al., 2018a), can effectively control the expression of the inflammation-related signaling molecule NLRP3 and thereby regulate the inflammatory pathway. In brief, the MAPK family consists of three major serine/threonine kinase proteins, namely, JNK, ERK and p38. Previous studies have shown that members of the MAPK family are activated after cisplatin exposure (Malik et al., 2015). The phosphorylation of MAPK exacerbates renal function and further causes apoptosis and inflammation (Pabla and Dong, 2008). Here, we discovered that farrerol substantially declined cisplatin-induced MAPK phosphorylation and p-NF-κB activation *in vitro* (Figures 3C–E) and *in vivo* (Figures 8A–C). As described above, ROS can also induce the phosphorylation of ERK and p38 to regulate apoptosis *via* p53 (Martindale and Holbrook, 2002), which is a tumor-suppressor protein that plays a key role in the induction of apoptosis in various cell types (Jiang et al., 2007). Here, we detected the levels of p53, Bax, caspase-3, and Bcl2 by Western blot analysis and found that the expression levels of p-p53, Bax, and caspase-3 were significantly increased in the cisplatin group and that Bcl2 expression was increased after pretreatment with farrerol (Figures 4A,B,D,E). In addition, TUNEL staining (Figures 4C,D) and flow cytometry (Figures 4E,F) assays indicated that farrerol evidently alleviated apoptosis compared with cisplatin treatment alone. Therefore, the scavenging of ROS by farrerol could prevent cisplatin-induced p-p53 activation and effectively decrease MTECs apoptosis. In short, we concluded that farrerol can alle*via*te the cisplatin-induced inflammatory response and apoptotic pathway by inhibiting ROS production *in vitro* and *in vivo*.

## Conclusion

**Figure 10:**
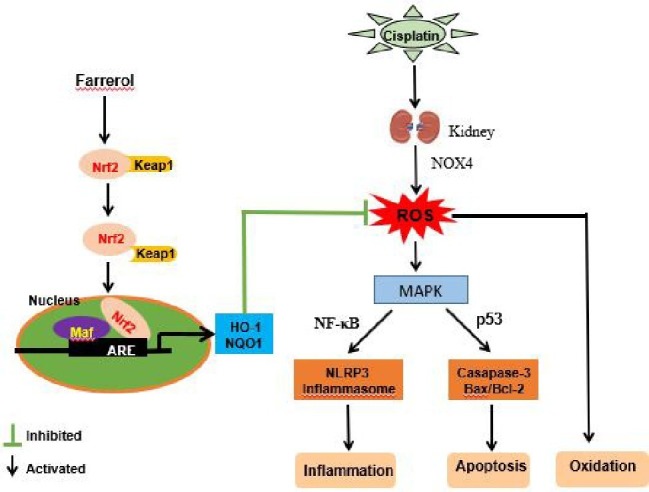
Protective effects of farrerol on CDDP-induced AKI and the underlying mechanism. Cisplatin enters the kidney and induces the production of a large amount of ROS. On the one hand, ROS can directly cause oxidative stress, and on the other hand, ROS activate NF-κB and p53 *via* the MAPK pathway and thereby induce changes in inflammation and apoptosis. Farrerol alleviates the generation of ROS by activating Nrf2, which in turn regulates the oxidation, inflammation, and apoptosis signaling pathways.

In summary, our study demonstrated that farrerol can improve cisplatin-induced nephrotoxicity by ameliorating the oxidative, inflammatory, and apoptotic pathways (Figure 10). This mechanism of kidney protection further alleviates the generation of ROS by activating Nrf2, which in turn regulates the signaling pathways associated with oxidation, inflammation, and apoptotic factors. These experiments demonstrated that farrerol, as a natural Nrf2 activator, has potential for the treatment of clinical AKI.

## Data Availability Statement

All datasets analyzed for this study are included in the article/supplementary material.

## Ethics Statement

The animal study was reviewed and approved by the Animal Health and Research Ethics Committee of Jilin University.

## Author Contributions

NM, WW, and XC designed the study. NM, WW, and XF performed the study. XF and XC helped in analyzing the data. NM and WW wrote the manuscript.

### Conflict of Interest

The authors declare that the research was conducted in the absence of any commercial or financial relationships that could be construed as a potential conflict of interest.
